# Artificial Intelligence and Robotics in Minimally Invasive and Complex Surgical Procedures: A Systematic Review

**DOI:** 10.7759/cureus.81339

**Published:** 2025-03-28

**Authors:** Eman Ibrahim Abdalla Osman, Manasik M. ElMurtada Mubarak Ismail, Mohamed Ahmed Hassan Mukhtar, Ahmed Umballi Babiker Ahmed, Nihal Ahmed Abd Elfrag Mohamed, Ali Ahmed Alamin Ibrahim

**Affiliations:** 1 Surgery, Hafar Albatin Central Hospital, Hafar Al-Batin, SAU; 2 General Surgery, Pilgrim Hospital, Boston, GBR; 3 Emergency Medicine, Najran Armed Forces Hospital, Ministry of Defense Health Services, Najran, SAU; 4 General Surgery, Najran Armed Forces Hospital, Ministry of Defense Health Services, Najran, SAU; 5 Obstetrics and Gynecology, Armed Forces Hospital, Wadi Al Dawasir, SAU; 6 Surgery, Nile University, Khartoum, SDN

**Keywords:** artificial intelligence, complex surgeries, minimally invasive surgery, robotics, surgical precision and efficiency

## Abstract

Robotics and artificial intelligence (AI) are transforming surgery by improving patient outcomes, efficiency, and precision. The use of robotics in surgery tackles important issues, including surgical precision, minimally invasive procedures, and healthcare accessibility, as global healthcare systems embrace AI-driven technologies more and more. However, access gaps and ethical issues with automation continue to exist on a worldwide scale, calling for a fair discussion of these developments. The aim of this systematic review was to summarize the most recent literature on the role of AI and robotics in minimally invasive and complex surgical procedures. The study was conducted as per Preferred Reporting Items for Systematic Reviews and Meta-Analyses (PRISMA) guidelines. We search for relevant studies across four different databases (PubMed, Scopus, Web of Science, and Google Scholar). We also restricted our search to 2024 to capture the most recent advancement only. We found 393 relevant studies, of which only 12 were included in this study upon assessing them with inclusion and exclusion criteria. The review ensures scientific rigor and openness by evaluating surgical specializations, AI technologies, and outcomes such as accuracy, recovery, and complications. The results show significant progress in AI-powered surgical systems, enhancing judgment, lowering surgical errors, and enabling individualized treatment plans. AI-enhanced visualization, real-time data processing, and automated robotic equipment are notable innovations that boost patient safety and procedure efficiency. The importance of these developments is emphasized throughout the discussion, especially with regard to developing minimally invasive procedures and increasing surgical capabilities for complex surgeries. However, obstacles to broad adoption are cited, including expenses, moral dilemmas, and the requirement for stringent training procedures.

## Introduction and background

The integration of artificial intelligence (AI) in surgical procedures has transformed modern healthcare, offering unprecedented precision, efficiency, and patient outcomes [1,2]. AI-driven technologies, including machine learning, deep learning, and robotics, are increasingly being adopted in minimally invasive and complex surgical procedures to enhance decision-making, optimize surgical planning, and improve intraoperative navigation [3]. These advancements have led to reduced complications, shorter recovery times, and improved surgical accuracy, making AI a valuable asset in modern surgical practice [4].

Minimally invasive surgery (MIS) refers to procedures performed with minimal tissue disruption, utilizing techniques such as laparoscopy, endoscopy, and robotic-assisted surgery [5]. Compared to traditional open surgery, MIS offers benefits such as reduced postoperative pain, shorter hospital stays, and lower infection rates. However, these procedures require exceptional precision and dexterity, which can be enhanced through AI-powered surgical assistance systems [6]. In complex surgical procedures, where precision is critical, such as in neurosurgery, cardiovascular surgery, and reconstructive surgery, AI aids in real-time decision support, robotic automation, and predictive analytics to optimize patient safety and outcomes [7].

Machine learning, a subset of AI, refers to algorithms that analyze patterns within vast datasets to improve decision-making over time, while deep learning, a more advanced form, uses artificial neural networks to process complex medical images and enhance surgical navigation [3]. For instance, AI-powered imaging tools assist in identifying tumors with higher accuracy in oncologic surgeries, while robotic-assisted laparoscopic platforms enhance dexterity in delicate procedures like prostatectomy [2]. Additionally, natural language processing (NLP) aids in automating surgical documentation, reducing administrative burdens on surgeons. These applications illustrate how AI technologies directly impact various aspects of modern surgery, ensuring both precision and efficiency in diverse clinical settings [6].

Recent advancements in AI-powered surgical robotics have further revolutionized the field. Systems such as the da Vinci Surgical System, AI-assisted navigation platforms, and computer vision-enhanced robotics allow surgeons to perform intricate procedures with superior accuracy [8]. AI algorithms analyze vast datasets, providing real-time feedback to improve intraoperative decision-making. Additionally, AI assists in preoperative planning, enabling surgeons to simulate procedures, predict potential complications, and tailor interventions based on patient-specific data [9].

Despite these promising developments, challenges remain in fully integrating AI into surgical practice. Ethical concerns, data privacy, regulatory constraints, and the need for clinical validation continue to pose barriers to widespread adoption. Moreover, variations in AI performance across different surgical specialties and settings necessitate further research to establish standardized protocols and assess the long-term impact of AI-driven interventions.

This systematic review aims to critically evaluate the current literature on the role of AI in minimally invasive and complex surgical procedures, identifying its benefits, limitations, and future implications. By synthesizing findings from diverse surgical specialties, this review will provide valuable insights into the evolving landscape of AI in surgery and its potential to redefine modern surgical practices.

## Review

Methodology

Protocol

This systematic review was conducted following the Preferred Reporting Items for Systematic Reviews and Meta-Analyses (PRISMA) guidelines to ensure a comprehensive and structured approach [10]. The methodology included a systematic search, study selection, data extraction, risk of bias assessment, and data synthesis.

Search Strategy

**Figure 1:**
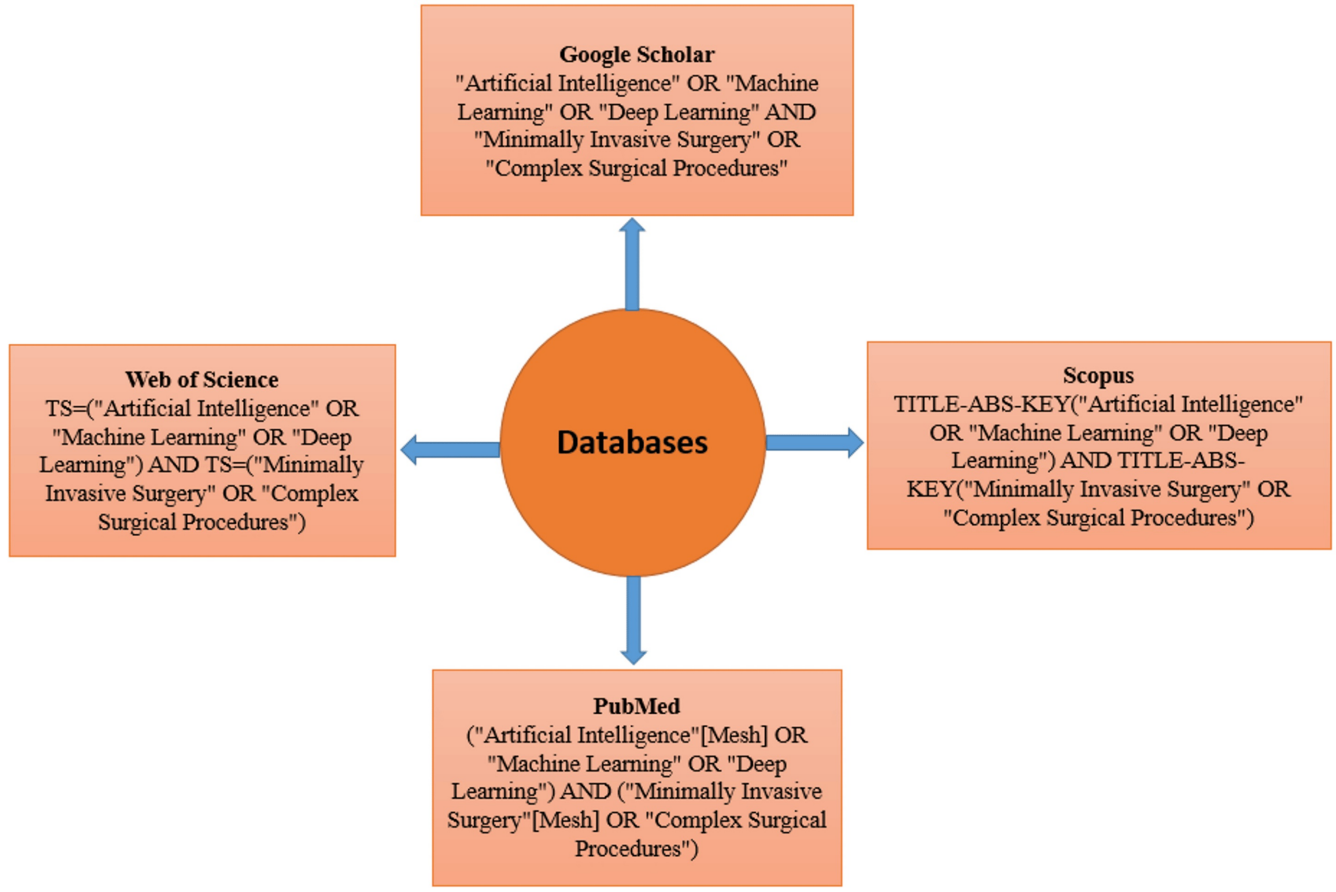
Search strings for PubMed, Scopus, Web of Science, and Google Scholar MeSH: Medical Subject Headings

A systematic literature search was performed across PubMed, Scopus, Web of Science, and Google Scholar to identify relevant studies on AI in minimally invasive and complex surgical procedures. The search strategy incorporated Medical Subject Headings (MeSH), keywords, and Boolean operators to ensure broad coverage while maintaining precision. The search was restricted to studies published in English within the last year (2024) to capture the most recent advancements in AI applications in surgery. Additionally, the reference lists of included studies were manually screened for further relevant articles. Figure 1 outlines the search strings for four different databases.

Eligibility Criteria

The eligibility criteria were predefined to ensure the inclusion of high-quality and relevant studies. Studies were included if they evaluated AI-based technologies such as machine learning, deep learning, robotics, or decision-support systems in minimally invasive or complex surgical procedures. Eligible study designs included clinical trials, observational studies, systematic reviews, and meta-analyses that reported on surgical precision, complication rates, efficiency, or patient recovery. Exclusion criteria included studies that focused solely on AI in diagnostics, non-surgical applications, or preoperative assessments, as well as opinion pieces, conference abstracts, and low-quality studies with a high risk of bias.

Studies Selection

Two independent reviewers from the list of authors, selected based on their expertise in AI and robotic-assisted surgery, screened the titles and abstracts of identified studies according to the inclusion criteria. Full-text articles were retrieved for further evaluation, and disagreements regarding eligibility were resolved through discussion or consultation with a third reviewer. The final set of included studies was documented using the PRISMA flow diagram, outlining the number of records identified, screened, included, and excluded at each stage.

Data Extraction

Data extraction was performed using a structured data extraction form to ensure consistency and accuracy. The extracted information included study details (author, year, country, study design), AI technology used, surgical specialty, type of procedure, outcome measures (surgical precision, complication rates, recovery time), key findings, and limitations. The process was conducted independently by two reviewers, with any discrepancies resolved through discussion.

Methodological Quality Assessment

To assess the methodological quality of the included studies, the Risk of Bias in Non-randomized Studies of Interventions (ROBINS-I) tool was applied. Each study was evaluated across seven domains, including bias due to confounding, selection of participants, classification of interventions, deviations from intended interventions, missing data, measurement of outcomes, and selection of the reported result. Studies were categorized as having low, moderate, or high risk of bias, ensuring a critical appraisal of their reliability and validity.

Data Synthesis

A narrative synthesis was conducted to summarize the findings across different AI applications in minimally invasive and complex surgical procedures. Due to heterogeneity in study designs, surgical specialties, and outcome measures, a quantitative meta-analysis was not performed. Instead, studies were grouped based on AI technology type (robotics, machine learning, decision-support systems), surgical specialty (general surgery, neurosurgery, urology, etc.), and outcome measures (surgical precision, complication rates, patient recovery, and efficiency). The synthesis highlighted key trends, benefits, limitations, and future implications of AI in surgery.

Results

Search Results

**Figure 2:**
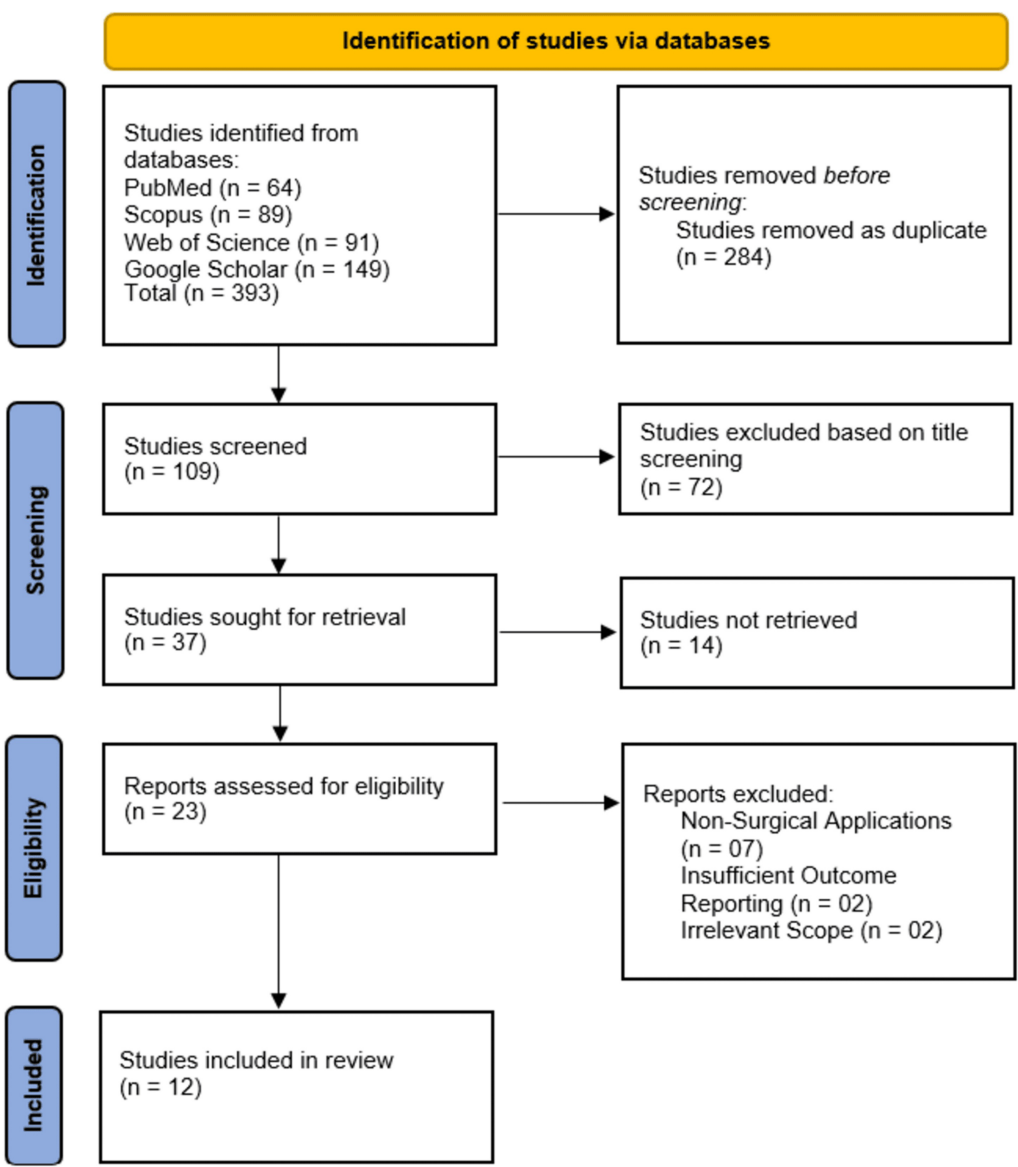
Preferred Reporting Items for Systematic Reviews and Meta-Analyses (PRISMA) flowchart

A total of 393 studies were identified from four major databases: PubMed (n = 64), Scopus (n = 89), Web of Science (n = 91), and Google Scholar (n = 149). After removing 284 duplicate records, 109 studies remained for title and abstract screening. Following this process, 72 studies were excluded based on relevance to the research question. The remaining 37 studies were sought for full-text retrieval, of which 14 could not be accessed due to availability issues. Subsequently, 23 full-text articles were assessed for eligibility. Of these, 11 studies were excluded for reasons including non-surgical applications (n = 7), insufficient outcome reporting (n = 2), and irrelevant scope (n = 2). Finally, 12 studies met the eligibility criteria and were included in this systematic review. The study selection process is detailed in the PRISMA flowchart (Figure 2).

Characteristics of Included Studies

This systematic review included 12 studies that explored the application of AI in minimally invasive and complex surgical procedures across various surgical specialties. The studies examined different AI-driven technologies, including robotics, digital imaging, machine learning, and AI-assisted decision-making systems, highlighting their impact on surgical precision, efficiency, and patient outcomes (Table 1).

**Table 1:** Characteristics and key findings of included studies AI: artificial intelligence; AI-driven robotics: artificial intelligence-driven robotics; AI in robotics: artificial intelligence in robotics; AI in surgery: artificial intelligence in surgery; AI-enhanced robotics: artificial intelligence-enhanced robotics; AI and robotics integration: artificial intelligence and robotics integration; digital imaging: digital medical imaging; MIS: minimally invasive surgery; RAS: robotic-assisted surgeries; GI surgeries: gastrointestinal surgeries; pediatric surgeries: pediatric surgeries; HNS: head and neck surgeries

Author and publishing year	AI technology	Surgical specialty	Surgical procedure	Outcome measures	Key findings	Limitations
Abbasi and Hussain (2024) [2]	AI-driven robotics	General surgery	Precision and efficiency	Efficiency and surgical accuracy	Demonstrates how AI may be used to increase surgical precision	Limited attention to particular surgical specializations
Abid et al. (2024) [11]	AI in robotics	urology	Urological surgeries	Recovery time and surgical outcomes	Focuses attention to AI's promise but needs clinical confirmation in the actual world	Mainly theoretical and narrow scope
Duong et al. (2024) [12]	AI in surgery	Plastic surgery	Reconstructive surgery	Patient satisfaction and surgical precision	AI can improve plastic surgery results in terms of appearance	Limited data of clinical trial
Farooq and Zahra (2024) [13]	Robotics and AI	Spine surgery	Minimally invasive spine surgery	Complication rates and surgical precision	Highlights the advantages of robotic technologies for spine surgery	Lacks data from extensive clinical studies
Liu et al. (2024) [14]	AI-enhanced robotics	General surgery	Various surgical procedures	Complication rates and surgical precision and surgical accuracy	Illustrates how AI in surgery has evolved	Lacking clinical results and concentrates on technology developments
Poh et al. (2024) [15]	AI, digital imaging, robotics	Ophthalmology	Vitreoretinal surgery	Surgical outcomes and patient recovery	Using AI and robotics together enhances ophthalmology surgery results	Limited range of AI types
Takeuchi and Kitagawa (2024) [16]	AI in surgery	Gastroenterology	Gastrointestinal surgeries	Risk minimization and surgical precision	AI enhances the results of gastrointestinal surgery	Absence of extensive clinical trials
Tejedor and Denost (2024) [17]	AI and robotics integration	General surgery	Robotic-assisted surgeries	Complication rates and surgical accuracy	Uses AI to increase accuracy	Narrow in scope and lacking of a thorough clinical assessment
Tsai et al. (2024) [18]	AI in surgery	Pediatric surgery	Pediatric surgeries	Complication rates and surgical outcomes	AI has potential in pediatric surgery	Absence of clinical studies and empirical data
Varghese et al. (2024) [19]	AI in surgery	General surgery	Various surgical procedures	Risk minimization and surgical accuracy	The revolutionary potential of AI in general surgery	Prioritizes technical and theoretical elements
Wojtera et al. (2024) [20]	AI in surgery	Head and neck surgery	Head and neck surgeries	Complication rates and surgical outcomes	AI enhances the results of head and neck operations	Insufficient evidence for clinical applicability
Zhang et al. (2024) [21]	AI in robotic surgery	General surgery	Robotic surgeries	Recovery time and surgical accuracy	Robotic surgical accuracy is increased with AI integration	Lacks in-depth clinical analysis and instead concentrates on narrative review

In terms of surgical specialties, AI was applied in general surgery [2,14,17,19,21], urology [11], plastic surgery [12], spine surgery [13], ophthalmology [15], gastroenterology [16], pediatric surgery [18], and head and neck surgery [20]. The reviewed studies assessed a variety of surgical procedures, including robotic-assisted surgeries, reconstructive procedures, minimally invasive spine surgery, vitreoretinal surgery, and gastrointestinal surgeries. The key outcome measures examined in these studies included surgical accuracy, complication rates, recovery time, and overall procedural efficiency.

The findings indicated that AI integration significantly improved surgical precision, minimized complications, and optimized patient recovery. Abbasi and Hussain [2], Liu et al. [14], and Zhang et al. [21] reported that AI-driven robotics enhanced accuracy in general surgery. Farooq and Zahra [13] highlighted the benefits of robotic assistance in minimally invasive spine surgery, while Poh et al. [15] demonstrated improved surgical outcomes in ophthalmology using AI-enhanced digital imaging. Additionally, Tsai et al. [18] and Wojtera et al. [20] emphasized AI’s potential in pediatric and head and neck surgeries, respectively, despite a lack of extensive clinical trials.

However, some studies, such as those by Abid et al. [11] and Varghese et al. [19], were primarily theoretical and lacked real-world validation. Several studies, including those by Duong et al. [12] and Takeuchi and Kitagawa [16], reported limited clinical data and called for larger-scale trials to confirm AI’s long-term impact. A common limitation across studies was the narrow scope of AI applications, with many focusing on technical advancements rather than direct clinical outcomes.

Overall, these studies provide valuable insights into AI’s transformative potential in surgery, reinforcing its benefits while also emphasizing the need for further clinical research, validation, and integration into standard surgical practices.

Methodological quality assessment results

The assessment revealed that four studies had a low risk of bias, indicating strong methodological quality and minimal threats to validity. The remaining eight studies were categorized as moderate risk of bias, primarily due to limitations such as insufficient clinical validation, moderate reporting bias, and a lack of extensive comparative analyses. Importantly, no studies were classified as high risk, suggesting that the included literature provides a reasonably reliable foundation for evaluating the role of AI in surgical procedures (Table 2).

**Table 2:** Methodological quality assessment using risk of bias in non-randomized studies of interventions (ROBINS-I) tool

Study	Bias due to confounding	Bias in selection of participants	Bias in classification of interventions	Bias due to deviations from intended interventions	Bias due to missing data	Bias in measurement of outcomes	Bias in selection of the reported result	Overall bias
Abbasi and Hussain (2024) [2]	Low	Low	Moderate	Low	Low	Moderate	Moderate	Moderate
Abid et al. (2024) [11]	Low	Low	Moderate	Low	Low	Moderate	Moderate	Moderate
Duong et al. (2024) [12]	Low	Low	Low	Low	Moderate	Low	Low	Low
Farooq and Zahra (2024) [13]	Moderate	Low	Low	Low	Moderate	Low	Low	Moderate
Liu et al. (2024) [14]	Low	Low	Moderate	Low	Low	Moderate	Moderate	Moderate
Poh et al. (2024) [15]	Low	Low	Low	Low	Low	Low	Low	Low
Takeuchi and Kitagawa (2024) [16]	Low	Low	Moderate	Low	Low	Low	Low	Low
Tejedor and Denost (2024) [17]	Low	Low	Moderate	Low	Moderate	Moderate	Moderate	Moderate
Tsai et al. [18]	Moderate	Moderate	Low	Low	Moderate	Moderate	Low	Moderate
Varghese et al. (2024) [19]	Low	Low	Low	Low	Low	Low	Low	Low
Wojtera et al. (2024) [20]	Low	Low	Moderate	Low	Moderate	Low	Low	Moderate
Zhang et al. (2024) [21]	Low	Low	Moderate	Low	Low	Moderate	Moderate	Moderate

Discussion

The use of robotics and AI in surgery has become a groundbreaking advancement that could greatly improve patient outcomes, surgical accuracy, and efficiency. As AI-powered technologies, such as ML, DL, and visual computing, continue to grow, their implementation into robotic systems is changing numerous surgical specialties, offering unparalleled advancements. The results of the recent literature are summarized in this systematic review, which shows notable advancements in robotic surgery in a variety of medical specialties, including spine surgery, ophthalmology, urology, and plastic surgery.

Advancements in AI-Driven Robotic Surgery

It has been demonstrated that incorporating AI into automated devices improves outcomes by reducing human error, ensuring higher precision, and enabling more intricate procedures. This is especially important for delicate surgeries like urology and aesthetic surgery, where doctors can make extremely accurate decisions in real time with the help of AI-enabled robotic equipment [2]. Additionally, by automating repetitive activities, AI-powered robots can lessen tiredness and free up surgeons to concentrate on more important areas of the process, improving overall efficiency and results. For example, the use of AI-powered robots in spine surgery has improved recovery times and reduced problems by enabling more precise screw placement [14].

Similarly, with enhanced imaging and navigation abilities, AI and robotic technologies have played a significant role in ophthalmology, enabling high-precision therapies such as robotic-assisted vitreoretinal surgery, automated retinal disease detection, and AI-driven laser treatments for conditions like diabetic retinopathy and age-related macular degeneration. Notwithstanding these developments, there are still issues, mostly related to cost, accessibility, and the requirement for intensive training to guarantee that medical personnel are capable of using these complex systems [13]. The high expense of AI-driven robotic systems continues to be a major deterrent to their broad use, especially in environments with limited resources. Additionally, integrating AI into surgery requires a thorough understanding of the technological advances, necessitating extensive instruction for surgeons to work with AI systems in an efficient manner [19].

Ethical Considerations and Challenges

The ethical issues with AI in surgery are also highlighted in the paper, including the possibility of an excessive dependence on technology and its effects on the connection between the surgeon and the patient. To guarantee patient safety and the moral application of new technologies, human oversight must continue to be at the center of surgical decision-making, even though AI can improve surgical precision [13,17]. AI-driven robotics in surgery has a bright future ahead of it, as research continues to enhance the technology's precision, safety, and adaptability. AI has the potential to completely transform surgery, not just by enhancing surgical results but also by democratizing healthcare by lowering the cost of accurate, effective, and high-quality procedures globally [2].

Thus, even though there are still obstacles to overcome, there is a great deal of promise for improving patient experiences, advancing medical care, and building a more effective healthcare system through the continuous integration of robotics and AI into surgical practice. According to the reviewed research, AI-driven robots may establish themselves as a mainstay of contemporary surgical practice with additional development and improvement, leading to improved results and a wider range of superior surgical procedures [2,12,21].

Impact on Surgical Workflow

In a variety of specialties, including urology, aesthetic surgery, spine surgery, ophthalmology, and pediatric surgery, AI technologies, such as machine learning, deep learning, and computer vision, have made impressive strides in surgical robotics, greatly increasing precision and improving patient outcomes. Research by Liu et al. [14] and Zhang et al. [21] shows how AI algorithms can enhance the decision-making powers of robotic systems, allowing for more accurate incisions, better tissue detection, and real-time surgical corrections. AI is now positioned as a viable tool for both complex and less invasive surgical operations due to these developments, which also result in lower complication rates, quicker recovery periods, and increased patient satisfaction. For instance, the potential of AI to improve precision has resulted in fewer surgical mistakes and a simpler approach to complicated surgeries, like those performed in pediatric and plastic surgery, where delicate, high-risk procedures are frequently performed. Furthermore, it has been demonstrated that integrating AI-driven robotic systems lowers the risk of infection, hemorrhage, and surgical errors during surgery. Varghese et al. [19] claim that AI systems provide real-time feedback, track vital signs, and are able to detect possible dangers before they become serious. This is particularly important for high-risk procedures like spine and head and neck surgery, where AI enhances surgical precision in spinal fusion, optimizes tumor resection planning, and improves real-time navigation in complex head and neck reconstructions.

Better patient results are eventually a result of these skills, which guarantee increased accuracy and safety. The literature also highlights how AI might improve the efficiency of surgical workflow [13,17]. Preoperative planning, intraoperative navigation, and postoperative monitoring are all aided by AI-powered systems, which add up to quicker and more precise treatments. These enhancements are especially helpful in clinical settings with high patient volumes, where efficiency in time is essential for lowering healthcare expenses and enhancing overall quality of service. AI-driven robotic devices help to improve the safety and availability of surgical interventions by cutting down on procedure time and improving operational precision. However, there are still obstacles in the way of the broad use of AI in surgery, even with the encouraging advantages [11,22].

Accessibility is still severely limited by the expensive nature of robotic systems, particularly in environments with limited resources. Furthermore, a recurring issue is that surgeons must have extensive training to operate this sophisticated equipment. Takeuchi and Kitagawa [16] have pointed out that even if AI-driven robotics is developing quickly, human supervision is still essential to guaranteeing the security and effectiveness of surgery. To guarantee that AI technologies are applied properly and morally and to avoid relying too much on machines for important judgments, surgeons must continue to actively participate in decision-making processes. To guarantee that AI is utilized as an auxiliary tool rather than a replacement, ethical issues, including the possible loss of individual discretion and the significance of AI in replacing specific human functions, must also be carefully considered [18]. Therefore, even if AI-driven robotic systems have an opportunity to completely transform surgery, continued cooperation between trained surgeons and AI technologies is necessary to optimize the advantages and reduce the hazards of these cutting-edge systems [15,23].

Key Recommendations

The application of AI to surgical robotics offers a thrilling chance to transform healthcare systems around the world, but in order to realize its full potential, a few crucial suggestions are necessary. First, to guarantee the efficient and secure use of AI-driven robotics, standard instructions and protocols are essential. Surgeons and other medical personnel should receive thorough training that addresses both the clinical decision-making needed to collaborate with AI and the technological aspects of running robotic systems. This would guarantee that medical professionals could successfully incorporate AI into their work while upholding strict safety and care guidelines.

Second, accessibility and cost-effectiveness continue to be major obstacles to the widespread use of robotic systems driven by AI, especially in environments with limited resources. Many healthcare facilities, especially those in underdeveloped nations, cannot afford these technologies due to their hefty upfront costs. To lower the cost of these technologies, governments and healthcare systems should look into funding options like grants, subsidies, and partnerships. Furthermore, assessing AI's long-term cost-effectiveness in robotic surgery is crucial to comprehending its total worth, taking into account not just the operational expenses but also the possibility of improved patient outcomes, lower rates of complications, and quicker recovery periods.

Third, a thorough discussion of the ethical issues pertaining to AI use in surgery is required. The function of AI when making decisions and the degree of human oversight necessary should be clearly defined by set norms. To promote public trust and confidence in emerging technologies, ethical issues, including data privacy, informed consent, and the possibility of AI-driven prejudices, must be addressed. The development and application of AI systems must respect moral principles and give patients' welfare top priority. Furthermore, for AI to be successfully incorporated into surgical practice, interdisciplinary cooperation between surgeons, AI specialists, engineers, and medical professionals is essential. To guarantee that AI systems satisfy clinical requirements while upholding ethical standards, interdisciplinary teams ought to be established. Additionally, cooperation will ensure that AI-driven solutions are safe, efficient, and easy to use in clinical settings by bridging the gap between technological research and real-world implementation.

Lastly, the advancement of AI and robotics in surgical practice depends on ongoing research and innovation. The goal of ongoing research should be to increase the safety, accuracy, and adaptability of AI-driven robotic systems, especially in high-stakes procedures where the highest level of precision is required. Future studies should also look into how AI might be used in cutting-edge surgical specialties like personalized surgery or regenerative medicine, where it could improve surgical results even more. AI-driven surgical robotics will be more fully utilized by healthcare systems if these technologies are continuously innovated and improved.

Implications

While integrating AI-driven robotics into surgery has the potential to be transformative, there are also significant obstacles that must be overcome to guarantee fair access and the greatest possible advantages. In addition to tackling ethical issues like algorithmic bias and patient privacy, policymakers are essential in developing rules that guarantee the safe and efficient application of AI technologies [13]. To ensure that employees can fully utilize AI-driven solutions, healthcare organizations must make investments in the education and upskilling of healthcare workers. In order to reduce inequalities in access to cutting-edge surgical treatment, infrastructure support is crucial, particularly in underprivileged areas [11].

It is imperative that technology developers concentrate on developing affordable, easily navigable solutions based on AI that can be smoothly integrated into a range of surgical specialties, such as ophthalmology, spine surgery, plastic surgery, and urology. For healthcare results to be equitable, it is imperative that these technologies be available to a wider range of healthcare practitioners, particularly in settings with limited resources. To overcome these obstacles and create a healthcare environment that optimizes the advantages of AI-driven surgical innovations, cooperation between legislators, medical facilities, and developers is also crucial [16].

Limitations

Despite providing a thorough summary of recent studies on AI-driven surgical robotic systems, the study has a number of shortcomings that should be noted. First, the evaluation contained a large number of observational studies, which means that they are susceptible to the biases that come with non-randomized designs. More high-caliber randomized controlled trials (RCTs) are obviously required to show a causal relationship between the incorporation of AI technologies and better surgical results, as well as to thoroughly assess the clinical efficacy of AI-driven robotic systems. The holes in current sources of evidence would be filled by RCTs, which would offer more convincing proof of the long-term advantages, dangers, and effectiveness of these advancements. Furthermore, a major drawback of the examined studies is the absence of long-term monitoring data, which limits our comprehension of the long-term effects of AI-driven robotics. The long-term impacts on patient health, expenses, and possible adverse effects are yet unknown and need more research, even if the short-term advantages, such as lower complication rates and quicker recovery times, have been thoroughly documented. The diversity of the reviewed papers is another drawback. Regarding the AI technologies utilized, surgical specialties addressed, and outcome metrics used, the studies included in the review differ greatly from one another. It is difficult to directly compare study results because of these variations.

There were differences in the ways that success was assessed, such as decreased rates of complications, accuracy, or recovery durations, between studies that examined AI applications in urology and those that investigated its usage in spine surgery, as well as ophthalmology. It would be easier to make more insightful comparisons and contribute to the development of a more cohesive body of data about the efficacy of AI in robotic surgery if outcome measures and research procedures were standardized across studies. Additionally, the evaluation did not thoroughly examine the larger healthcare system and infrastructural factors that can influence the acceptance and integration of these technologies, instead concentrating primarily on the implementation of AI in robotic surgery. How successfully AI-driven robotics may be used in various healthcare settings depends on a number of factors, including hospital rules, regulatory frameworks, resource availability, and the degree of experience of medical experts. To give a more thorough picture of the opportunities and difficulties associated with implementing AI technology in surgery, future studies should take these larger contextual elements into account. Lastly, the evaluation mostly uses research from high-income nations, which can restrict how broadly the results can be applied in low- and middle-income contexts where access to cutting-edge medical technology is frequently limited. These areas' limited healthcare expenditures, lack of infrastructure, and inadequate medical professional training may all have an impact on the adoption of AI-driven robotics. In order to better comprehend the global usefulness of these technologies, future research should fill this knowledge gap by examining the viability, obstacles, and results of AI-driven robotics in countries with low or middle incomes. This would guarantee that the advantages of AI in surgeries can be expanded to various healthcare environments, fostering fair access to cutting-edge surgical care across the globe.

## Conclusions

Modern medicine is changing as a result of the incorporation of robotics and AI in surgical practice, which offers significant improvements in accuracy, productivity, and patient outcomes. Numerous fields have shown the encouraging potential of AI-driven robotic systems in improving surgical precision and lowering risks, including plastic surgery, urology, spine surgery, head and neck surgery, and ophthalmology. The evolution of AI in surgery continues to advance, optimizing procedures and expanding its applications across different medical fields. Additionally, AI’s role in improving surgical outcomes is becoming increasingly evident in areas such as vitreoretinal diseases and pediatric surgery. However, challenges remain, including the need for standardized protocols, further clinical validation, and addressing ethical considerations in AI adoption. With continuous advancements in machine learning algorithms and robotic systems, the future of AI in surgery appears promising. Ongoing research and development will be crucial in unlocking the full potential of AI, ensuring better surgical outcomes, and reducing overall healthcare costs.
